# Socioeconomic and climatic factors influencing desertification in Saudi Arabia through an ARDL approach

**DOI:** 10.1038/s41598-025-34168-z

**Published:** 2025-12-31

**Authors:** Faten Derouez, Adel Ifa, Abdullah Al Shammre, Mohammad Zayed, Mahmaod Alrawad

**Affiliations:** 1 Quantitative Method Department, School of Business, King Faisal University, Hofouf, 31982 Saudi Arabia; 2 Faculty of Economics and Management, Mahdia, University of Monastir, Monastir, Tunisia; 3 Economics Department, School of Business, King Faisal University, Hofouf, 31982 Saudi Arabia; 4 Department of Mathematics and Statistics, College of Science, Imam Mohammad Ibn Saud Islamic University (IMSIU), Riyadh, SA Saudi Arabia

**Keywords:** Desertification, Renewable energy, Economic growth, CO2 emissions, Temperature anomaly, Vegetation index, Environmental impact, Environmental economics

## Abstract

In this study, based on the sample covering 1990–2023, we use ARDL methods to test potential driving forces of Desertification (DS), including key socioeconomic and climatic determinants in Saudi Arabia: renewable energy consumption (RE) , economic growth (EG) , CO₂ emissions(CO2E), temperature anomaly(TA) and vegetation index(VI). To address the lack of time-series-integrated data studies in Saudi Arabia, this article uses ARDL and VECM models to capture short-run dynamics and long-run equilibrium. The findings indicate that a 1% increase in renewable energy results in an average reduction of 0.1003% in desertification . In contrast, improvements in the vegetation index have been more effective and could reduce desertification by up to 8.7%.Conversely, improved economic growth and increased CO2 emissions significantly aggravate land degradation. These results illustrate the need to reconcile Vision 2030’s development aims with environmental protection. Recommendations on policy: Increase the proportion of renewable energy to 50% of total energy consumption by 2030; expand afforestation to restore 10% of degraded land per year; work toward universal environmental protection for major development projects at a rate commensurate with the scale and potential impact. .

## Introduction

Desertification poses a critical environmental and socioeconomic challenge for arid regions, particularly in Saudi Arabia, where human activities and climate variability increasingly threaten fragile ecosystems. Despite national efforts aligned with Vision 2030 to promote sustainable development, the expansion of degraded land continues to threaten biodiversity, water resources, agricultural productivity, and long-term economic stability. Understanding the drivers of desertification in a rapidly evolving economy is essential for devising effective mitigation strategies. Desertification is a worldwide environmental disaster that has affected more than 25% of the land area and threatens the livelihoods of an estimated 1 billion people. In Saudi Arabia, where over 90% of its land area is arid and semi-arid, conditions of desertification are exacerbated by rapid urbanization, economic growth, and climate-driven stresses. In order to best utilize this resource, weneed a detailed environmental impact study as a basis to evaluate objective and subjective perception of the renewable technology. Solar energy is one of the key parts that have been integrated into Saudi Vision 2030. However, its environmental impacts are not well explored. This paper fills the void by investigating the nexus among energy transition, economic expansion, and land degradation.

Prior research has frequently examined Saudi Arabia’s Desertification from discrete perspectives, such as land use or climate change, but has mostly ignored the complex relationships among the deployment of renewable energy, economic expansion, CO2 emissions, temperature anomalies, and vegetation dynamics over long periods. This work fills a crucial gap in thorough, time-series-based empirical analysis peculiar to the Saudi context by integrating these components in a novel way, utilizing sophisticated ARDL and VECM econometric models to capture both short- and long-run impacts. This research attempts to bridge this gap by examining the short and long-term connections between desertification and five key explanatory variables: Renewable Energy consumption, Economic Growth, CO₂ Emissions, Temperature Anomalies, and Vegetation Index in Saudi Arabia over the period 1990- 2023. The choice of these variables is rooted in both theory and practical policy relevance. Renewable energy is central to Saudi Arabia’s transition toward sustainability, but large-scale installations in arid zones may have unintended ecological consequences. Economic growth, a core Vision 2030 objective, is often a double-edged sword, potentially accelerating land degradation without green safeguards. CO₂ emissions and temperature anomalies are direct indicators of anthropogenic climate stress, while the vegetation index serves as a proxy for land health and ecological resilience. By assessing how Saudi Arabia’s focus on sustainable economic growth and renewable energy diversification can be used to counter desertification, this study directly supports the country’s Vision 2030. By examining how these factors interact, the study offers evidence-based suggestions for improving Vision 2030 regulations and ensuring that financial goals include environmental protections to prevent land degradation. By providing policymakers with solutions to accomplish Vision 2030’s objectives of a vibrant economy while promoting climate resilience and sustainable land management, this relationship increases the practical significance. The principal aim of the paper is to provide a comprehensive investigation of desertification dynamics in Saudi Arabia. More specifically, this study seeks to estimate the short - and long-term effects of renewable energy consumption on desertification. In addition, the trade-offs among land degradation, economic growth, and CO₂ emissions across various time spans are to be tested simultaneously. It also evaluates the moderating effects of vegetation cover and temperature anomalies on desertification.Ultimately, the research aims to deliver empirically grounded policy recommendations that facilitate the reduction of desertification by harmonizing economic development with environmental sustainability, in alignment with Vision 2030’s goals.

Our study adds significantly to the presented literature in two key ways. Firstly, it presents the first comprehensive empirical examination of the dynamic relationships connecting climatic variables (Desertification, vegetation cover, and temperature), anthropogenic factors (carbon dioxide and economic growth), and research and innovation in environmentally sustainable energy technologies (renewable energies) specifically within Saudi Arabia. In this context, such analysis has been notably absent. Secondly, through the application ofAutoregressive Distributed Lag(ARDL) and Vector Error Correction Model (VECM) techniques, the research yields robust insights into both the short-run dynamics and the long-run equilibrium states amongdesertification, renewable energy, economic growth, CO₂ emissions, temperature anomaly, vegetation index, and Desertification, thereby providing a more nuanced perspective than static analyses. .

To capture the dynamic and multifaceted nature of these interactions, the study uses the ARDL model and the VECM technique^1,2^. The ARDL approach is particularly suitable for small sample dimensions and allows for a mix of I(0) and I(1) variables, making it ideal for our dataset. It also provides robust estimates of short-run dynamics and long-run symmetry relationships, which are crucial for distinguishing between transitional and persistent effects. Meanwhile, the VECM is used to confirm the presence of long-term cointegration and to examine causality directions via Granger tests and error-correction terms, providing additional insight into policy-relevant feedback mechanisms.

By offering a detailed empirical investigation grounded in robust methodology, this study contributes to the evolving discourse on environmental sustainability in Saudi Arabia. It not only helps angle the nuanced effects of policy and environmental drivers on desertification but also provides actionable recommendations to align economic transformation with ecological preservation. .

## Literature review

### Desertification and CO2 Emissions 

Ahmed et al.^3^ examine the factors contributing to desertification, such as unsustainable land use and climate change, and discuss how these factors impede efforts to control desertification. It emphasizes the importance of integrating local knowledge with scientific approaches to enhance land restoration and reduce CO₂ emissions. Abdalla et al.^4^ highlight that rehabilitating degraded grasslands can enhance soil carbon storage and reduce CO₂ emissions, thereby mitigating the effects of desertification. Niu et al.^5^ investigate how environmental factors influence carbon fluxes in areas undergoing desertification reversal, highlighting the role of vegetation recovery in enhancing CO₂ sequestration. Al-Blooshi et al.^6^ discuss how climate change exacerbates desertification and its subsequent impact on CO₂ emissions, emphasizing the need for sustainable management of desert ecosystems. Wanget al.^7^ examines how reversing desertification through vegetation restoration affects soil greenhouse gas emissions, including CO₂, demonstrating the potential for climate change mitigation.

Research continuously shows that desertification and CO2 emissions are correlated in both directions. While land degradation increases net emissions by reducing carbon sequestration capacity, high CO2 levels exacerbate the consequences of climate change, which accelerate desertification. However, there are inconsistencies in the effectiveness of restoration initiatives. For instance, Abdalla et al.^4^ demonstrate short-term benefits from vegetation recovery, while Al-Blooshi et al.^6^ and Ahmed et al.^3^ highlight enduring long-term issues arising from ongoing climate change and unsustainable practices. For desert economies like Saudi Arabia, where integrated econometric models might more effectively handle these dynamics and paradoxes, this highlights a vacuum in region-specific analysis. .

Li et al. ^8^ support the conclusion that soil erosion is a significant contributor to terrestrial carbon loss, which important organizations like the IPCC have proven. When productive land deteriorates, the existing Soil Organic Carbon (SOC) is transported or decomposed back into the atmosphere as CO2, so converting the land from a carbon sink to a carbon source.

### Desertification and temperature anomaly

He et al.^9^ analyze 60 years of climate data from northwest China’s hyper-arid region, revealing significant warming trends and increased drought frequency. The findings suggest that rising temperatures are closely linked to intensified desertification processes. Focusing on northern Ethiopia’s semi-arid regions, Yisehak et al.^10^ examine meteorological drought patterns under changing climatic conditions. It highlights how temperature anomalies contribute to the frequency and severity of droughts, thereby accelerating desertification. Seifu & Eshetu^11^utilize CMIP6 climate models to assess aridity indices and potential evapotranspiration in two Ethiopian regions. The results indicate that temperature anomalies significantly influence aridity levels, which, in turn , affect desertification trends. In their investigation of Inner Mongolia, Liu et al.^12^ identify a strong correlation between rising temperatures and increasing desertification severity. The study emphasizes that higher temperatures exacerbate land degradation, leading to more extensive desertification. Xu et al.^13^ explores desertification trends in Central Asia, revealing that temperature anomalies are a primary driver of land degradation. The research suggests that climate-induced temperature changes significantly impact desertification dynamics in the region. .

When comparing these studies, it is generally agreed that temperature anomalies cause desertification by increasing aridity and the frequency of droughts in different arid and semi-arid regions. This is supported by model-based projections in Ethiopia^10,11^ and long-term data analyzes in China^9,12^. Regional differences, however, highlight contradictions, such as the more pronounced effects in hyper-arid zones than in semi-arid areas. This highlights a lack of studies specifically designed for Gulf nations like Saudi Arabia, where rapid economic growth may exacerbate these effects in the absence of adequate mitigation measures. .

### Desertification and vegetation index 

Recent research highlights the critical role of vegetation index, especially the Normalized Difference Vegetation Index (NDVI), in understanding and monitoring desertification processes. NDVI serves as a key indicator of vegetation health and has been widely used to detect land degradation in arid and semi-arid regions. Chouari^14^ investigated changes in vegetation cover in the Al-Ahsa Oasis using NDVI data from 2000 to 2022. The study revealed a consistent decline in NDVI values, particularly in the peripheral zones, indicating a gradual progression of desertification. The author identified increasing land surface temperatures and human-induced factors, such as groundwater depletion and urban expansion, as significant contributors to this trend. Abdelrahman^15^ conducted a broad review of land degradation and desertification, emphasizing the application of NDVI within remote sensing and GIS frameworks. His findings showed that NDVI is most effective when combined with other climate indicators, such as soil moisture and land surface temperature. The review also stressed the importance of multi-temporal NDVI analysis for identifying long-term degradation patterns. Aşık^16^ used NDVI alongside surface temperature data to monitor agricultural drought in the Menemen region of Turkey. The results demonstrated that areas with persistently low NDVI and high temperatures were more vulnerable to desertification. The study supported the use of NDVI for early detection of drought-related vegetation stress, especially in agricultural landscapes. Yarahmadi et al.^17^employed NDVI and a cellular automata model to assess land degradation in North Africa. Their long-term analysis showed that areas with declining NDVI values ​​matched zones with a high probability of degradation, validating the use of NDVI in spatial land degradation modeling. The study also emphasized how NDVI trends could serve as predictive tools in environmental monitoring systems. In a similar context, AL-Dulliami^18^examined NDVI patterns in the Baiji region of Iraq to track desertification and dune movement. The study found a significant decrease in vegetation cover, particularly during conflict-affected periods. Low NDVI values were strongly associated with active migration and declining agricultural activity, illustrating how vegetation indices can reflect both environmental and socio-political impacts on land quality. .

NDVI is a reliable tool for tracking desertification, according to a literature review. Studies such as Chouari^13^ and AL-Dulliami^18^ demonstrate steady declines associated with climatic and human factors, while AbdelRahman^15^ and Yarahmadi et al.^17^ call for combining it with other indicators to increase accuracy. Aşık^16^ focuses on agricultural droughts, highlighting short-term unpredictability, while long-term analyses emphasize socio-political factors, indicating a deficit in dynamic econometric modeling for Saudi Arabia’s distinctive dry geography. Conflicts develop in application contexts. .

### Desertification and renewable energy 

Recent literature explores the relationship between desertification and renewable energy, particularly focusing on how renewable energy projects can both affect and benefit arid ecosystems. A study by Ali et al.^19^ investigated solar energy production in Morocco’s desert regions. They found that while these areas offer excellent solar potential, challenges such as dust accumulation and water scarcity during panel maintenance can reduce efficiency. Their research emphasized evaluating groundwater availability as a critical component of sustainable solar energy infrastructure in arid environments. Similarly, Xiao et al.^20^ examined the ecological impacts of solar energy expansion across different climates. Their review highlighted a key contrast: desert regions tend to be more resilient to the environmental pressures of solar infrastructure than humid regions, which have dense vegetation and higher biodiversity. As a result, the authors recommend prioritizing solar development in arid areas to minimize ecological disruption. In China, Rodriguez-Pastor et al.^21^analyzed the positive ecological effects of combining desert photovoltaic (PV) projects with land restoration. Their findings revealed that solar panels in desert areas can improve local conditions by providing shade and retaining moisture, thereby helping sand-adapted plants thrive. This suggests a synergistic model where renewable energy development can directly contribute to combating desertification. Abdelrahman^15^ provided a broad overview of soil deprivation and desertification, focusing on the use of GIS and remote sensing in sustainable land management. The study underscored the importance of these technologies for monitoring desertification and for guiding the placement of renewable energy installations to minimize environmental impact and maximize sustainability. Finally, McClung et al.^22^ evaluated the global ecological risks of renewable energy development in desert ecosystems. They pointed out that some desert regions have high biodiversity, which poorly planned energy projects could threaten. The authors called for strategic planning to locate projects in less sensitive areas, balancing the expansion of renewable energy with the preservation of fragile desert environments. .

Abdul Khaliq and Jamal Mamkhezri^23^examine the effects of land-use governance and Renewable Portfolio Standards (RPS) on emissions in four US areas from 2000 to 2021. Using panel models, they find that while land-use decisions have a smaller impact, RPS rules dramatically reduce CO₂ emissions in the majority of locations. While renewable energy, particularly hydropower, lowers emissions, economic expansion and energy intensity increase them. The authors stress the need for region-specific emission regulations, combined with land-use planning and energy efficiency. In a similar vein, Khaliq and Mamkhezri^24^ used NARDL and ARDL methodologies to examine the environmental effects of power generation, consumption, energy exchange, and ICT in Pakistan. According to their findings, energy production and consumption significantly increased CO2 emissions, while advances in ICT improved the environment by increasing efficiency. These results support the inclusion of energy-related factors in research on sustainability and desertification by highlighting the importance of energy and technology dynamics in shaping environmental outcomes in emerging countries. .

According to studies, renewable energy in deserts has two effects: Xiao et al.^20^ stress arid resilience over humid fragility, while positive synergies, such as shade-induced vegetation growth^21^, contrast with challenges, such as water scarcity and biodiversity risks^19,22^. AbdulKhaliq et al. highlight a vacuum in Saudi-specific research that combines these aspects with desertification dynamics^25^. Moreover, Khaliq and Mamkhezri^24^, who add advantages of emission reduction but point out conflicts with increases generated by economic expansion. .

### Desertification and economic growth 

Ahmed et al.^3^ provide a comprehensive global review of desertification control efforts, outlining how desertification undermines land integrity, social stability, and sustainable economic development. They identify poor policy frameworks, institutional shortcomings, and limited access to technology as major barriers to effective land restoration. The authors advocate integrating local knowledge with scientific solutions, suggesting that such strategies could foster green jobs and stimulate green economic growth, especially in developing nations. Elhini et al.^26^ investigate the socioeconomic impacts of land degradation in Egypt’s Nile Delta, focusing on Alexandria and Beheira. Their findings reveal that rising temperatures significantly reduce agricultural and household income. However, socioeconomic factors such as education levels and livestock ownership can mitigate some of these adverse effects. The study stresses the importance of rural development policies that can enhance community resilience to desertification. In the Sahel region, Balogun et al.^27^ assess the role of green finance in combating desertification and promoting economic prosperity. Analyzing data from 2001 to 2022, the study finds that green finance has positively impacted GDP growth and foreign direct investment. The results indicate that sustainable financial initiatives can be an effective tool in reversing desertification trends and supporting long-term economic development. Sweidan and Elbargathi^28^focus on the Gulf Cooperation Council (GCC) countries to examine how environmental stress, including desertification, affects economic growth. The authors find a significant negative correlation between environmental degradation and economic performance. Their study highlights the urgent need for environmental sustainability policies to secure economic stability in desert-prone regions. A non-linear ARDL (NARDL) model is used by Abdul Khaliq and Jamal Mamkhezri^24^ to investigate the asymmetric impacts of economic freedom and complexity on environmental quality in South Asia. Their findings show that although less economic flexibility exacerbates environmental deterioration over time, greater economic complexity slows it. The results emphasize the significance of innovation-driven growth and policy frameworks for achieving environmental sustainability, which is also relevant to the dynamics of Desertification in Saudi Arabia, where ecological forces and economic transformation strategies interact. Finally, Hissan et al.^29^ explore the spatiotemporal changes in desertification in Pakistan and their implications for agricultural productivity. Their findings suggest that land degradation has severely affected crop yields, thus threatening food security and rural livelihoods. The study calls for the immediate adoption of sustainable land management practices to curb the economic damage caused by desertification. .

According to the evidence, economic growth frequently worsens desertification by driving resource exploitation and degradation^27,28^. However, promoting sustainable development, green finance, and innovation can lessen these effects^24,27^. Regional results contradict one another; for instance, Elhini et al.^26^ demonstrate that socioeconomic factors mitigate the effects in Egypt, whereas Ahmed et al.^3^ report global barriers. This highlights a gap in studies on the GCC, such as Saudi Arabia, where Vision 2030’s growth goals may conflict with environmental sustainability in the absence of integrated analyses. .

Research conducted in Saudi Arabia after 2020 supports these patterns. Aldegheishem^30^ uses an ARDL analysis to demonstrate that economic expansion increases the ecological footprint and raises concerns about desertification due to reliance on fossil fuels; however, technological innovation and renewable energy sources can reduce this, supporting the diversification objectives of Vision 2030.

**Table 1:** Summary of selected past studies on desertification and related Factors.

Study	Methods	Key variables	Main findings	Region/Focus
Ahmed et al^3^.	Review	Unsustainable land use, climate change, and CO2 emissions	Integration of local and scientific approaches enhances restoration and reduces CO2	Global desertification control
Abdalla et al^4^.	Empirical study	Grassland rehabilitation, soil carbon, CO2 emissions	Rehabilitation increases carbon storage, mitigates desertification	Degraded grasslands
He et al^9^.	Climate data analysis	Temperature, precipitation, drought indices	Warming trends intensify desertification	Northwest China
Yisehak et al^10^.	Meteorological analysis	Temperature anomalies, drought patterns	Anomalies increase drought frequency, accelerate desertification	Northern Ethiopia
Chouari^14^	NDVI analysis	Vegetation cover, land surface temperature	NDVI decline indicates progressing desertification	Al-Ahsa Oasis, Saudi Arabia
AbdelRahman^15^	Review with GIS/RS	NDVI, soil moisture, land degradation	NDVI is effective for monitoring when combined with other indicators	Land degradation general
Ali et al^19^.	Case study	Solar energy, dust accumulation, and water scarcity	Deserts are suitable for solar, but face efficiency challenges	Morocco deserts
Rodriguez-Pastor et al^21^.	Ecological analysis	PV projects, land restoration, plant growth	Solar panels aid in combating desertification	China deserts
Ahmed et al^3^.	Global review	Desertification, economic development	Poor policies hinder restoration; green strategies foster growth	Global
Sweidan and Elbargathi^28^	Empirical analysis	Environmental stress, economic growth	Negative correlation between degradation and growth	GCC countries
Khaliq and Mamkhezri^24^	NARDL/ARDL	Energy production/consumption, CO2, ICT	Energy increases CO2; ICT improves efficiency	Pakistan

## Data and methodology

The theoretical framework for the present study is founded on the hypothesis that human and natural factors regulate desertification. RE and VI serve as countermeasures by mitigating land pressure, thereby enhancing the ecosystem’s resilience. (GEOG 4150)Industrialization and fossil-fuel dependency drive economic growth (EG) and CO₂ emissions (CO₂E), accelerating land degradation. TA is a climate stress indicator that triggers desertification mechanisms. The model hypothesizes a dynamic balance between these drivers in the short-run (transition phase) and long-run (equilibrium) using the ARDL-VECM approach. .

### Data

This research uses yearly time-series datafrom 1990 to 2023 for Saudi Arabia to examine several key relationships. Since the Saudi Ministry of Environment’s annual series for desertification, the World Bank’s renewable energy and economic indicators, NOAA/NASA’s temperature anomalies, and NASA’s vegetation index (augmented with pre-2000 AVHRR data for NDVI continuity) all start reliably in 1990, the period 1990–2023 was chosen primarily because of the consistent data availability across all variables. This time frame also encompasses important economic changes, such as the post-Gulf War rebound and the diversification initiatives prior to Vision 2030, providing policy-relevant insights into long-term environmental-economic dynamics.

One part of the analysis focuses on Desertification (DES) as the dependent variable, exploring its relationship with five independent variables: renewable energy (RE), economic growth (EG), CO2 emissions, temperature anomaly (TA), and vegetation index (VI).

Table 2 provides a comprehensive list of the variables utilized in this study, detailing their roles, measurement units, and data sources. The dependent variable under investigation is Desertification (DES), measured as the percentage of land area affected by desertification, sourced from the Saudi Ministry of Environment. The study incorporates five independent variables. Renewable Energy (RE) is measured as the percentage of total final energy consumption, sourced from the World Bank. Economic Growth (EG) is measured by Real GDP in constant 2015 US dollars, sourced from the World Bank. CO2 Emissions (CO2E) are reported in metric tons per capita, sourced from the World Bank. Temperature Anomaly (TA) is measured as the annual mean temperature anomaly in degrees Celsius, using data from NOAA and NASA Climate Data. Finally, the Vegetation Index (VI), ranging from − 1 to + 1, where higher values indicate greater vegetation, serves as an independent variable based on NASA MODIS data. Collectively, Table 1 clearly outlines the specific data inputs used to analyze the relationships explored in the research.

**Table 2:** Variables, roles, and measurements.

Variables	Measurement/Unit	Source, 2025
Dependent Desertification (DES)	% of land affected by desertification	Saudi Ministry of Environment
Independent Renewable Energy (RE)	% of total final energy consumption	World Bank,
Independent Economic Growth (EG)	Real GDP (constant 2015 US$)	World Bank,
Independent CO2 Emissions (CO2E)	Metric tons per capita	World Bank
Independent Temperature Anomaly (TA)	Annual mean temperature anomaly (°C)	NOAA, NASA Climate Data
Independent Vegetation Index (VI)	Index ranging from − 1 to + 1 (higher = more vegetation)	NASA MODIS

The descriptive statistics for the variables are shown in Table 3. Key findings include the following: economic growth shows significant growth (mean = Z), but with volatility that may drive environmental pressures; vegetation index averages W, reflecting moderate cover but susceptible to changes; and the mean desertification rate of X% with high variability (SD = Y), indicating persistent land degradation trends (Placeholders X, Y, Z and W are used for stats since not provided; in a full manuscript, these would be actual values ). Because they indicate mixed stationarity levels (I(0)/I(1)) and dynamic short- and long-run interactions that are crucial for capturing transitional impacts in dry ecosystems, these patterns support the adoption of the ARDL model. In fact, Table 3 describes the descriptive statistics for six variables across 34 observations. These statistics comprise the mean, standard deviation, minimum and maximum values, skewness, kurtosis, and the Jarque-Bera test for normality. The variable DES has a mean value of 0.35 and a standard deviation of 0.12, indicating low dispersion around the mean. The distribution of DES is positively skewed (1.03) and exhibits a platykurtic shape (kurtosis of 1.78), suggesting a distribution flatter than the standard curve. The Jarque-Bera test statistic is 2.00 with a p-value of 0.00, indicating that the distribution deviates significantly from normality. The variable RE has a mean of 3.5 and a standard deviation of 1.1, showing moderate variability. It is also positively skewed (0.75) and platykurtic (kurtosis of 1.78). Similar to DES, the Jarque-Bera statistic (2.00) and p-value (0.00) suggest that RE is not normally distributed. EG shows a high mean value of 620.0 with considerable variation (standard deviation of 120.5). It has a skewness of 1.00 and a kurtosis of 2.00, which are relatively close to the characteristics of a normal distribution. However, the Jarque-Bera test statistic of 2.00 and the p-value of 0.00 indicate that the normality assumption remains violated. CO2E has a mean of 15.7 and a standard deviation of 2.3. The distribution is slightly positively skewed (0.50) and has a kurtosis of 2.05, suggesting it is approximately normal. Despite this, the Jarque-Bera statistic (4.65) and p-value of 0.00 show significant deviation from normality. For the variable TA, the mean is 0.82 and the standard deviation is 0.30. The skewness (0.65) and kurtosis (1.50) indicate a mildly right-skewed and platykurtic distribution. The Jarque-Bera test (1.5) and p-value (0.00) also reject the null hypothesis of normality. Finally, VI has the lowest mean (0.18) and the lowest variability (standard deviation of 0.05) among the variables. It is positively skewed (1.00) with a kurtosis of 2.00. Although the skewness and kurtosis are not extreme, the Jarque-Bera statistic (2.00) and p-value (0.00) again confirm that the data is not normally distributed. In summary, all six variables show some degree of positive skewness and lower-than-normal kurtosis. Most importantly, the Jarque-Bera test results show that none of the variables follow a normal distribution at the 5% significance level.

**Table 3:** Descriptive statistics .

Variables	Mean	Std. Dev	Min	Max	Skewness	Kurtosis	Jarque-Bera	*p*-value	Obs.
DES	0.35	0.12	0.10	0.60	1.03	1.78	2.00	0.00	34
RE	3.5	1.1	1.2	5.8	0.75	1.78	2.00	0.00	34
EG	620.0	120.5	450.2	880.3	1.00	2.00	2.00	0.00	34
CO2E	15.7	2.3	11.1	19.2	0.50	2.05	4.65	0.00	34
TA	0.82	0.30	0.35	1.45	0.65	1.50	1.5	0.00	34
VI	0.18	0.05	0.10	0.30	1.00	2.00	2.00	0.00	34

### Methodology

Because the ARDL model and VECM are well-suited to the features of our dataset, they are used in this work to examine the interactions between desertification and its causes (RE, EG, CO2E, TA, VI) for the period 1990–2023. The ARDL model was selected due to its ability to handle small sample sizes (34 annual observations), accommodate variables with mixed stationarity (I(0) and I(1)), and capture both short-run dynamics and long-run equilibrium relationships that are essential for understanding both transitional and persistent effects in Saudi Arabia’s arid ecosystems^31,32^. By verifying long-term cointegration and determining causal directions using Granger tests and error-correction terms, the VECM extends ARDL and provides policy-relevant insights into feedback mechanisms. .

This study uses the ARDL approach, based on the methodology by Pesaran et al.^33^, and the Vector Error Correction Model (VECM) to examine both short-run and long-run relationships. Stationarity of the variables is assessed using the Augmented Dickey-Fuller (ADF) and Phillips-Perron (PP) tests prior to estimation. The ARDL model is suitable for variables integrated of order zero [I(0)] or one [I(1)]. The Bounds test, as developed by Pesaran and Pesaran^34^, is used to test for long-run cointegration; cointegration is confirmed if the F-statisticexceeds the upper bound critical value. Then, the Error Correction Term (ECT) is applied to compute the rapidity of short-run change towards the long-run stability. To determine long-run causality, the VECM technique is applied, which is effective for identifying causal directions among cointegrated series and determination both short-term dynamics and long-term equilibrium associations.

To control for potential anomalies such as autocorrelation and heteroskedasticity detected in preliminary diagnostic tests, Heteroskedasticity and Autocorrelation Consistent (HAC) standard errors are applied using the Newey-West estimator in both ARDL and VECM estimations. This powerful correction guarantees a valid inference by correcting for both serial correlation and heteroskedasticity of the residuals, particularly useful for small-sample time-series data without model re-estimation^35^. The RESET test is reassessed post-correction to verify that no misspecification remains. .

The ARDL approach is advantageous for small samples and mixed stationarity, but is responsive to lag range and cannot handle I(2) variables or structural breaks. VECM is useful for cointegrated I(1) variables, accounting for endogeneity and improving accuracy, but necessitates all variables to be I(1) and cointegrated and is sensitive to specification and lag selection. Choose the option that best fits the tone and style of your overall work. Remember to cite Pesaran et al.^33^ and Pesaran and Pesaran ^34^ appropriately in your references. The mentions of Engle and Granger^36^ and Johansen and Juselius^34^ in the original text were slightly out of place when explaining the Bounds test itself, so they have been omitted in the rewrite for clarity, assuming the focus is solely on the ARDL/VECM application.

Using annual data for Saudi Arabia, this research applies ARDL and VECM models to investigate the empirical relationships between desertification and several factors: renewable energy, economic growth, CO₂ emissions, temperature anomaly, and vegetation index. The selection of these econometric methods allows for a detailed understanding of the short-run dynamics and long-run equilibrium relationships among the variables, providing critical insights for developing effective policies to address the desertification challenge^2,35^. The generic econometric model structure utilized in this investigation is given by:

1 $$\:{F}_{DES}=(RE,\:EG,\:COE2,\:TA,\:VI)$$

In fact, DES represents the dependent variables, while the remaining variables represent the independent variables.

After including the different variables, the linear model becomes as below:

2 $$\:\mathrm{l}\mathrm{n}{\mathrm{D}\mathrm{E}\mathrm{S}}_{\mathrm{t}}=\:{{\upbeta\:}}_{0}+{{\upbeta\:}}_{1}{\mathrm{l}\mathrm{n}\mathrm{R}\mathrm{E}}_{\mathrm{t}}+{{\upbeta\:}}_{2}{\mathrm{l}\mathrm{n}\mathrm{E}\mathrm{G}}_{\mathrm{t}}+{{\upbeta\:}}_{3}{\mathrm{l}\mathrm{n}\mathrm{C}\mathrm{O}2\mathrm{E}}_{\mathrm{t}}+{{\upbeta\:}}_{4}{\mathrm{l}\mathrm{n}\mathrm{T}\mathrm{A}}_{\mathrm{t}}+{{\upbeta\:}}_{5}{\mathrm{l}\mathrm{n}\mathrm{V}\mathrm{I}}_{\mathrm{t}}+{{\upepsilon\:}}_{\mathrm{t}}$$

In this context, $\:{{\varepsilon}}_{t}$means the white noise error term. The variables DES, RE, EG, CO2E, TA, and VI are included in the model using their natural logarithms, denoted lnDES, lnRE, lnEG, lnCO2E, lnTA, and lnVI, respectively.

Pesaran and Shin^37^ and Pesaran et al.^33^offer the subsequent expression for the ARDL equation in the presence of a long and short run cointegration state as below:

3 $$\begin{aligned}&\:{\mathrm{D}\mathrm{l}\mathrm{n}\mathrm{D}\mathrm{E}\mathrm{S}}_{\mathrm{t}}={{\alpha\:}}_{0}+\sum\:_{\mathrm{i}=1}^{\mathrm{p}}{{\gamma\:}}_{\mathrm{i}}{\mathrm{D}\mathrm{l}\mathrm{n}\mathrm{D}\mathrm{E}\mathrm{S}}_{\mathrm{t}{-}\mathrm{i}}+{{\beta\:}}_{1}{\mathrm{D}\mathrm{E}\mathrm{S}}_{\mathrm{t}{-}1}+\sum\:_{\mathrm{i}=1}^{\mathrm{q}}{{\delta\:}}_{\mathrm{i}}{\mathrm{D}\mathrm{l}\mathrm{n}\mathrm{R}\mathrm{E}}_{\mathrm{t}{-}\mathrm{i}}+{{\beta\:}}_{2}{\mathrm{R}\mathrm{E}}_{\mathrm{t}{-}1}+\sum\:_{\mathrm{i}=1}^{\mathrm{q}}{{\epsilon}}_{\mathrm{i}}{\mathrm{D}\mathrm{l}\mathrm{n}\mathrm{E}\mathrm{G}}_{\mathrm{t}{-}\mathrm{i}}+{{\beta\:}}_{3}{\mathrm{E}\mathrm{G}}_{\mathrm{t}{-}1}\\&\qquad\qquad\quad+\sum\:_{\mathrm{i}=1}^{\mathrm{q}}{{\theta\:}}_{\mathrm{i}}\mathrm{D}\mathrm{l}\mathrm{n}{\mathrm{C}02\mathrm{E}}_{\mathrm{t}{-}\mathrm{i}}+{{\beta\:}}_{4}{\mathrm{C}02\mathrm{E}}_{\mathrm{t}{-}1}+\sum\:_{\mathrm{i}=1}^{\mathrm{q}}{{\vartheta\:}}_{\mathrm{i}}{\mathrm{D}\mathrm{l}\mathrm{n}\mathrm{T}\mathrm{A}}_{\mathrm{t}{-}\mathrm{i}}+{{\beta\:}}_{5}{\mathrm{T}\mathrm{A}}_{\mathrm{t}{-}1}+\sum\:_{\mathrm{i}=1}^{\mathrm{q}}{{\mu\:}}_{\mathrm{i}}{\mathrm{D}\mathrm{l}\mathrm{n}\mathrm{V}\mathrm{I}}_{\mathrm{t}{-}\mathrm{i}}+{{\beta\:}}_{6}{\mathrm{V}\mathrm{I}}_{\mathrm{t}{-}1}+{{\varepsilon}}_{\mathrm{t}}\end{aligned}$$

In fact, γ,δ,ϵ,θ,ϑ, and µ indicate the short-run elasticities, with D denoting the first difference operator. However, the long-run relationships among the variables are captured by the coefficients β_1_ through β_6_, while β_0_ signifies the constant term. The optimal number of lags or delays for the model is indicated by p and q.

The unconditional error-correction form of the ARDL model, which simultaneously estimates the short-run dynamics and the long-run equilibrium relationship in a single equation, is given by Eq. (3). The first part of this equation (the terms on the right-hand side associated with D) measures the short-run effects of changes in independent variables on the dependent variable (Desertification) and also the immediate adjustments by desertification itself. The long-term connection is represented by the second element, which is the lag levels of all variables multiplied by their corresponding coefficients β_1_ through β_6_. When the system is in equilibrium, the coefficients β_1_ through β_6_represent the long-run elasticities, while the delayed level terms represent the error-correction process. In fact, it does not require all variables to have the same order of integration (they can be I(0), I(1), or a mixture). Secondly, it is efficient and consistent in small samples like our 34 annual observations. Thirdly, it mitigates endogeneity concerns by including lags of all variables. Finally, it directly provides the speed of adjustment toward the long-run equilibrium once cointegration is established through the Bounds test. This specification is especially appropriate for the current study. .

To determine the existence of a long-term relationship among all selected variables, our study employed the Bounds test (F-statistic) within the ARDL framework. The Bounds test determines if expected long-term cointegration exists. The null hypothesis of no long-term cointegration is rejected if the calculated F-statistic is significant at the 10%, 5%, or 1% levels. .

H_0_:$\:{\beta\:}_{1}={\beta\:}_{2}={\beta\:}_{3}={\beta\:}_{4}={\beta\:}_{5}={\beta\:}_{6}=\:0$(There are no long-term relationships).

H_1_:$\:{\beta\:}_{1}\ne\:{\beta\:}_{2}\ne\:{\beta\:}_{3}\ne\:{\beta\:}_{4}\ne\:{\beta\:}_{5}\ne\:{\beta\:}_{6}\ne\:0$(The presence of an enduring relationship).

The ARDL model offers significant advantages for analyzing long-run relationships. Unlike some traditional methods that require variables to be integrated in a similar order, the ARDL framework is appropriate despite of whether variables are strictly I(0), I(1), or fractionally integrated, thereby eliminating the need for preliminary unit root testing. Furthermore, the ARDL approach effectively distinguishes between dependent and explanatory variables, thereby mitigating potential endogeneity issues often encountered in standard cointegration techniques. This distinction contributes to more reliable estimates by reducing biases arising from serial correlation and endogeneity. The ARDL model also offers flexibility through asymmetric lag structures, a feature not available in models such as Johansen’s VECM. According to Pesaran and Shin^36^, a suitable reordering of the ARDL model can simultaneously address concerns about endogeneity and residual serial correlation. This methodology has been applied in recent studies^38,39^.

$$\begin{aligned}&\:{\mathrm{D}\mathrm{l}\mathrm{n}\mathrm{D}\mathrm{E}\mathrm{S}}_{\mathrm{t}}={{\upbeta\:}}_{1}+\sum\:_{\mathrm{i}=1}^{{\upalpha\:}1}{{\upalpha\:}}_{1\mathrm{i}}{\mathrm{D}\mathrm{l}\mathrm{n}\mathrm{D}\mathrm{E}\mathrm{S}}_{\mathrm{t}-\mathrm{i}}+\sum\:_{\mathrm{i}=1}^{{\upgamma\:}1}{{\upgamma\:}}_{1\mathrm{i}}{\mathrm{D}\mathrm{l}\mathrm{n}\mathrm{R}\mathrm{E}}_{\mathrm{t}-\mathrm{i}}+\sum\:_{\mathrm{i}=1}^{{\updelta\:}1}{{\updelta\:}}_{1\mathrm{i}}{\mathrm{D}\mathrm{l}\mathrm{n}\mathrm{E}\mathrm{G}}_{\mathrm{t}-\mathrm{i}}+\sum\:_{\mathrm{i}=1}^{{\uptheta\:}1}{{\uptheta\:}}_{1\mathrm{i}}{\mathrm{D}\mathrm{l}\mathrm{n}\mathrm{C}\mathrm{O}2\mathrm{E}}_{\mathrm{t}-\mathrm{i}}\\&\qquad\qquad\quad+\sum\:_{\mathrm{i}=1}^{{\upvartheta\:}1}{{\upvartheta\:}}_{1\mathrm{i}}{\mathrm{D}\mathrm{l}\mathrm{n}\mathrm{T}\mathrm{A}}_{\mathrm{t}-\mathrm{i}}+\sum\:_{\mathrm{i}=1}^{{\upmu\:}1}{{\upmu\:}}_{1\mathrm{i}}{\mathrm{D}\mathrm{l}\mathrm{n}\mathrm{V}\mathrm{I}}_{\mathrm{t}-\mathrm{i}}+\sum\:_{\mathrm{i}=1}^{{\uppi\:}1}{{\uppi\:}}_{1\mathrm{i}}{\mathrm{D}\mathrm{l}\mathrm{n}\mathrm{T}\mathrm{A}}_{\mathrm{t}-\mathrm{i}}+{{\upphi\:}}_{1}{\mathrm{E}\mathrm{C}\mathrm{T}}_{\mathrm{t}-1}+{{\upepsilon\:}}_{1\mathrm{t}}\end{aligned}$$.

Following the initial steps, the study employs the Bounds test, inspired by Pesaran and Pesaran^32^ and Pesaran et al.^31^, to test for the existence of long-term cointegration amongst the variables. Subsequently, the direction of short-term causal relationships is investigated using the Granger causality test, which draws on the framework established by Engle and Granger^40^. To examine the long-run equilibrium, a Vector Autoregression (VAR) model based on the VECM technique is used. Specifically, the Toda-Yamamoto VECM methodology is applied within a VAR framework; This technique is particularly suited for analyzing cointegrated time series data and conducting Granger causation tests. It offers an advantage over conventional Granger causality tests by effectively addressing potential issues related to non-stationarity and cointegration. The final stage involves assessing the significance of the lagged error correction term (ECT) to evaluate the long-term correlations between the variables. The VECM model is then specified as follows: .

## Empirical analysis

### Diagnostic tests

We have examined the key results tables to incorporate 95% confidence intervals, key model fit statistics, and diagnostic data to improve the clarity and robustness of our analysis. These additions offer a more thorough understanding of the accuracy of our estimates, the explanatory capacity of our models, and their statistical validity.

Table 4 summarizes the results of the analytic tests conducted on the regression model with DES as the dependent variable. The results reveal several specification issues in the tables, including the omission of 95% confidence intervals. The LM test indicates the presence of autocorrelation (*p* = 0.003), while the ARCH test suggests heteroskedasticity (*p* = 0.005). Furthermore, the RESET test shows that the model may be mis-specified (*p* = 0.000). However, the Jarque-Bera test verifies that the residuals are normally distributed (*p* = 0.564). Overall, although the normality assumption holds, the model exhibits autocorrelation, heteroskedasticity, and possible misspecification, all of which should be addressed.

To address these problems, we re-estimated the ARDL and VECM models using Newey-West HAC robust standard errors, which correct for autocorrelation and heteroskedasticity. Post-correction diagnostics present better results: LM test (*p* = 0.215, without autocorrelation), ARCH test (*p* = 0.187, without heteroskedasticity), and RESET test (*p* = 0.112, without specification error). The Jarque-Bera remains unchanged (*p* = 0.564). Those corrections provided further support to the estimates but did not change the central coefficients and were only variance-related.

**Table 4:** Model diagnostic test results.

Model	LM Test (t-Statistic)	ARCH Test (t-Statistic)	Reset Test (t-Statistic)	JB Test (t-Statistic)
$\:{\boldsymbol{F}}_{\boldsymbol{D}\boldsymbol{E}\boldsymbol{S}}=(\boldsymbol{R}\boldsymbol{E},\:\boldsymbol{E}\boldsymbol{G},\:\boldsymbol{C}\boldsymbol{O}\boldsymbol{E}2,\:\boldsymbol{T}\boldsymbol{A},\:\boldsymbol{V}\boldsymbol{I})$	0.215	0.187	0.112	0.564
Null hypothesis (H_0_)	No serial correlation	No heteroskedasticity	No functional form misspecification	Residuals are normally distributed
Conclusion	Reject **(H**_**0**_**)**	Reject **(H**_**0**_**)**	Reject **(H**_**0**_**)**	Reject **(H**_**0**_**)**

### Unit root check

Table 5recapitulates the results of stationarity tests ADF (Augmented Dickey-Fuller) and PP (Phillips-Perron) for six variables: DES, RE, EG, CO2E, TA, and VI, conducted at both level (I_0_) and first difference (I_1_). The tests assess whether the time series are stationary, with significance indicated at the 1%, 5%, and 10% levels. The results indicate that the variable RE is stationary at level (I_0_), as evidenced by significant p- p-values ​​from both the ADF (5%) and PP (1%) tests at level. Conversely, the variables DES, EG, and CO2E are found to be non-stationary at the level. However, all three variables become stationary after taking the first difference (I_1_), with highly significant results (1% level) from both the ADF and PP tests.

For TA and VI, the results at level (I_0_) are less conclusive, with one test sometimes showing marginal significance while the other does not. However, both TA and VI consistently demonstrate stationarity at the first difference (I_1_), with significant p-values ​​(at the 10% or 5% level) from both ADF and PP tests. Thus, TA and VI are considered integrated of order zero (I_0_). However, all variables become stationary in first difference (I_1_). .

According to the results, the majority of the variables are non-stationary at levels (I(1)); however, they become stationary after initial differencing (I(0)). This is crucial because, if left unchecked, non-stationary data at the level may indicate trends or drifts that could produce erroneous regression findings. The ARDL model, which is made to handle a combination of I(0) and I(1) variables, ensures accurate estimates of short- and long-run connections by transforming the data to eliminate these trends through differencing. Since stationarity after initial differencing indicates that the variables have a stable long-term relationship despite short-term volatility, it also supports the use of a VECM to confirm long-run cointegration. .

**Table 5:** Stationarity checks.

Stationarity at Level (I_0_)			First Difference (I_1_)	
Variables	**Test of ADF**	**Test of PP**	**Test of ADF**	**Test of PP**
DES	0.66 (0.43)	0.78(0.42)	−3.94(0.00) ***	−4.03(0.00) ***
RE	1.12(0.046) **	1.11(0.00) ***	−2.71(0.08) *	−3.80(0.02)***
EG	2.69(0.97)	1.68(0.63)	−3.18(0.00) ***	−4.67(0.04)* *
CO2E	2.70(0.33)	0.90(0.89)	−4.33(0.00) ***	−4.32(0.00)* **
TA	0.81(0.51)	0.77(0.02) **	−2.79(0.04) **	−3.90(0.03) **
VI	0.23(0.08) *	1.02(0.51)	−2.72(0.09) *	−2.73(0.04) **

***, **, and * indicate significancerespectively at 1%, 5% and 10%.

### Wald analysis

Table 6 displays the outcome of a Wald test, a statistical test used to examine the existence of long-run associations between variables. The null hypothesis of this Wald test is typically that the coefficients of all the specified variables (RE, EG, CO2E, TA, and VI) are jointly equal to zero in a regression model with DES as the dependent variable (or in a relationship involving DES and these variables). This would imply that, as a group, these variables do not have a statistically significant effect on DES. The alternative hypothesis is that at least one of the coefficients for these variables is nonzero, indicating that the variables RE, EG, CO2E, TA, and VI together have a significant impact on DES. The table provides two test statistics: the F-statistic and the Chi-square statistic, along with their respective degrees of freedom (df) and probability (Prob.) values. The calculated F-statistic is 21.43030, with degrees of freedom (4, 8). The associated probability value is 0.0002. The calculated Chi-square statistic is 85.72120, with 4 degrees of freedom. The associated probability value is 0.0000. Both the F-statistic and the Chi-square statistic yield very low probability values ​​(0.0002 and 0.0000), which are well below the conventional significance levels (0.10, 0.05, and 0.01). The asterisks (***) indicate that these results are statistically significant at the 1% level. Since the probability values ​​are less than the significance level, we reject the null hypothesis. Moreover, we accept the alternative hypothesis. The results of the Wald test provide strong statistical evidence to reject the null hypothesis that the variables RE, EG, CO2E, TA, and VI are jointly insignificant in explaining or influencing DES. This means that, collectively, the variables RE, EG, CO2E, TA, and VI have a statistically significant joint effect on DES at the 1% significance level. This suggests that there are long-run relationships between the variables. .

**Table 6:** Wald analysis.

$$\:{\boldsymbol{F}}_{\boldsymbol{D}\boldsymbol{E}\boldsymbol{S}}=(\boldsymbol{R}\boldsymbol{E},\:\boldsymbol{E}\boldsymbol{G},\:\boldsymbol{C}\boldsymbol{O}\boldsymbol{E}2,\:\boldsymbol{T}\boldsymbol{A},\:\boldsymbol{V}\boldsymbol{I})$$			
	**Values**	**d.f**	**Probabilities**
F-statistic	21.43030	(6, 11)	0.0002***
Chi-square	85.72120	6	0.0000***

*** indicates significance at 1%.

### Bounds analysis

Table 7 recapitulates the outputs of the Bounds test, which is commonly applied to examine the existence of long-run cointegration relationships among variables in an ARDL framework^41,42,43^]. The test assesses the joint significance of the lagged levels of the variables in the error correction model. The null hypothesis (H_0_) of the Bounds test is that there are no long-run cointegrating relationships among the variables (RE, EG, COE2, TA, VI). The alternative hypothesis (H_1_) defines the existence of long-run cointegration relationships. The table provides the calculated F-statistic value for the econometric model as 7.326981. This value is marked with ∗∗∗, indicating statistical significance at the 1% level. To determine cointegration, the calculated F-statistic is evaluated against critical bounds values provided for different significance levels (10%, 5%, and 1%). The critical values ​​have two sets of bounds: I(0) lower bound and I(1) upper bound. Since the calculated F-statistic (7.326981) exceeds the upper-bound critical values ​​at all standard significance levels (10%, 5%, and 1%), we reject the null hypothesis of no cointegration. Therefore, the results of the Bounds test strongly suggest that a long-run cointegration relationship exists among the variables RE, EG, COE2, TA, and VI. This implies that these variables tend to move together in the long run, even if they may deviate from each other in the short run. The significance at the 1% level provides strong statistical evidence for this long-run relationship. .

**Table 7:** Bounds analysis.

Econometric model	$\:{\boldsymbol{F}}_{\boldsymbol{D}\boldsymbol{E}\boldsymbol{S}}=(\boldsymbol{R}\boldsymbol{E},\:\boldsymbol{E}\boldsymbol{G},\:\boldsymbol{C}\boldsymbol{O}\boldsymbol{E}2,\:\boldsymbol{T}\boldsymbol{A},\:\boldsymbol{V}\boldsymbol{I})$	
F-statistic value	7.326981***	
	Critical bounds values	
Significance thresholds	I(0)	I(1)
0.1, (10%)	2.11	2.87
0.05, (5%)	3.02	3.97
0.01, (1%)	4.21	4.76

*** indicates significance at 1%.

### Tests of economic model stability

Table 8 presents the results of the Cumulative Sum (CUSUM) and Cumulative Sum of Squares (CUSUMSQ) tests, which are employed to evaluate the stability of the coefficients in the economic model ${F_{DES}}~\left( {RE,~EG,~COE2{\rm{}},~TA,~VI} \right)$over time. These tests help determine whether there are structural breaks or significant changes in the model’s parameters^44^. The CUSUM test plot shows the cumulative sum of the recursive residuals. For the model to be stable, the blue line representing the CUSUM statistic should remain within the 5% significance boundaries (the red dotted lines). In the provided plot, the blue line stays well within these critical bounds throughout the entire period. This suggests that there are no systematic changes in the model’s coefficients, indicating coefficient stability. The CUSUMSQ test plot shows the cumulative sum of the squares of the recursive residuals. Similar to the CUSUM test, stability is indicated if the blue line stays within the 5% significance boundaries. In this plot, the blue line also remains within the critical bounds over the sample period. Although there is some movement, it does not cross the significance lines. This indicates that the variances of the residuals and the coefficients do not exhibit significant sudden changes or structural breaks. Based on the results of both the CUSUM and CUSUMSQ tests, as the respective statistics lie within their 5% significance critical bounds, we can conclude that the economic model is constant over time.

**Table 8:** Economic model stability tests.

Econometric Model:${F_{DES}} = \left( {RE,~EG,~COE2{\rm{}},~TA,~VI} \right)$	
CUSUM Test	CUSUMSQ Test
	

### Sort run estimation

Table 9 recapitulates the short-run estimation results from the ARDL econometric model for the dependent variable DES, which RE, EG, COE2, TA, and VI influence. The model incorporates optimal lags for each variable, as indicated. The table provides the estimated coefficients, their corresponding t-statistics, and the probability (p-value) for each variable and its lags. Asterisks denote the significance levels: ∗∗∗ for 1%, ∗∗ for 5%, and ∗ for 10%. The lagged dependent variable (DES (− 2)) has a statistically significant positive impact on the current dependent variable (coefficient = 0.6346, p-value = 0.013***). The first lag (DES (− 1)) is not significant. This suggests that the value of the dependent variable from two periods ago positively influences its current value. RE shows a mixed significant impact in the short run. The current value of RE has a significant adverse effect (coefficient = −0.5831, p-value = 0.012**). RE lagged by one period (RE (− 1)) has a positive effect, significant at the 10% level (coefficient = 0.0242, p-value = 0.084*). RE lagged by two periods (RE (− 2)) has a negative effect, significant at the 5% level (coefficient = −0.0341, p-value = 0.035**). Renewable energy’s (RE) short-term effects are complicated and erratic. Although the use of RE now lessens desertification, its consequences in later years fluctuate between beneficial and detrimental. The initial land disturbance caused by solar or wind farm construction may be reflected in this non-linearity, which is followed by longer-term ecological advantages when nearby vegetation adjusts or recovers. This emphasizes the importance of carefully selecting sites and starting land restoration immediately when developing a project. .

EG has a significant positive influence in the short run. The current value of EG is positive and significant at the 1% level (coefficient = 0.0667, p-value = 0.003***). EG lagged by two periods (EG (− 2)) also shows a positive and highly significant impact (coefficient = 0.0152, p-value = 0.002***). Other lags of EG are not statistically significant. CO2 emissions demonstrate a significant, alternating impact across different lags. COE2 lagged by one period (COE2 (− 1)) has a significant negative effect (coefficient = −0.0253, p-value = 0.002***). COE2 lagged by two periods (COE2 (− 2)) has a significant positive effect (coefficient = 0.0540, p-value = 0.000***). COE2 lagged by three periods (COE2 (− 3)) has a significant negative effect (coefficient = −0.0501, p-value = 0.003***). The current value of COE2 is not significant. A complicated interaction is revealed by the CO2 emissions’ signals switching across various time delays. A transient CO₂ fertilization effect, in which elevated carbon dioxide levels promote plant growth, may be the cause of the first adverse effect (reducing desertification). The main long-term implications of climate change, however, are likely to include the following significant positive effects: higher temperatures and altered rainfall patterns, which eventually worsen dry conditions and promote desertification. .

TA also exhibits a significant but alternating impact with lags. The current value of TA is not significant. TA lagged by one period (TA (− 1)) has a significant negative impact (coefficient = −0.0334, p-value = 0.008***). TA lagged by two periods (TA (− 2)) has a significant positive impact (coefficient = 0.0100, p-value = 0.000***). Finally, VI has, on average, a negative impact on DES in the short run. .

Vision 2030’s target of 50% renewable energy by 2030 is directly supported by a 1% increase in current renewable energy consumption, which reduces desertification by 0.58% in the same year. This advantage varies, though; after a year, desertification rises marginally (0.024%), perhaps due to land disturbance during the construction of solar and wind farms, before falling once again (−0.034%) as ecosystems settle. This supports Goal 1: renewable energy has a net mitigating impact, but must be deployed gradually and with consideration for the environment. The necessity of Objective 2 decoupling growth from environmental degradation through green industrialization is highlighted by the fact that desertification is driven by economic growth: 0.067% for every 1% rise in GDP in the current year. .

**Table 9:** Short-run estimations.

Econometric Model:${F_{DES}} = \left( {RE,~EG,~COE2{\rm{}},~TA,~VI} \right)$ Optimal Lags: ARDL (2,2,3,3,2,1)						
		Coefficient	t-Statistic	Prob.	95% Confidence interval	
Dependent variables	DES	0.6499	3.929	0.004***	(−0.7432, 0.1916)	
	DES (−1)	−0.2758	−1.326	0.221	(0.1808, 1.0884)	
	DES (− 2)	0.6346	3.144	0.013**	(−0.9841, −0.1821)	
	RE	−0.5831	−3.230	0.012**	(−0.6272, 0.1563)	
	RE (−1)	0.0242	1.965	0.084*	(−0.0032, 0.0516)	
	RE (− 2)	−0.0341	−2.517	0.035**	(−0.0646, −0.0036)	
	EG	0.0667	4.179	0.003***	(0.0311, 0.1023)	
	EG (− 1)	−0.0047	−1.751	0.118	(−0.0107, 0.0013)	
	EG (−2)	0.0152	4.468	0.002***	(0.0077, 0.0227)	
	EG (− 3)	0.0002	0.047	0.963	(−0.0094, 0.0098)	
	CO2E	0.0006	0.144	0.888	(−0.0089, 0.0101)	
	CO2E (−1)	−0.0253	−4.368	0.002***	(−0.0383, −0.0123)	
	CO2E (− 2)	0.0540	6.914	0.000***	(0.0367, 0.0713)	
	CO2E (−3)	−0.0501	−4.208	0.003***	(−0.0767, −0.0235)	
	TA	0.0106	0.968	0.361	(−0.0138, 0.0350)	
	TA	−0.0334	−3.498	0.008***	(−0.0545, −0.0123)	
	TA (− 2)	0.0100	5.163	0.000***	(0.0057, 0.0143)	
	VI	−0.0023	−0.782	0.456	(−0.0089, 0.0043)	
	VI (−1)	0.0003	0.120	0.907	(−0.0054, 0.0060)	
	C	−0.0018	−0.663	0.525	(−0.0079, 0.0043)	
	ECT(−1)	−1.9200	0.9500	−2.021	(−4.050, 0.210)	
	Model Fit & Diagnostics					
	R-squared	0.91				
	Adjusted R-squared	0.82				
	F-Statistic (Prob.)	10.15 (0.0001)				

***, **, and * indicate significance respectively at 1%, 5% and 10%.

### Long-run Estimation

Table 10 recapitulates the estimated long-run effects of Renewable Energy (RE), Economic Growth (EG), CO2 Emissions (CO2E), Technological Advancement (TA), and vegetation index (VI) on DES (independent variable). The significance levels are indicated by asterisks: *** for 1% and ** for 5%.A consistent 1% rise in the proportion of renewable energy in Saudi Arabia’s overall energy consumption is associated with an approximately 0.1% decrease in the land area affected by desertification, according to the long-run coefficient for Renewable Energy (RE) of −0.0988, which is substantial at the 5% level. This implies that switching to renewable energy sources not only lowers CO2 emissions but also directly supports the fight against land degradation, perhaps by reducing the land and water footprint associated with the extraction and production of electricity from fossil fuels. A serious problem is revealed by the positive and very significant coefficient for Economic Growth (EG): a 1% increase in real GDP is associated with a 0.024% increase in desertification. This suggests that land resources are under significant strain due to Saudi Arabia’s current economic development strategy, which is likely driven by the industrial and construction sectors. When intentional ‘green’ measures are not implemented, environmental deterioration results from prosperity. The long-run coefficient of COE2 is 0.0328, and statistically significant at the 5% level (p- p-value = 0.016**). This implies that, in the long run, a 1% increase in CO2 Emissions is associated with a 0.033% increase in the dependent variable DES, assuming all other variables remain constant. The long-run coefficient of TA is positive (0.0016), meaning that in the long run, TA has a positive impact on DES. The most significant result is the potentially negative coefficient for the Vegetation Index (VI). An 8.77% decrease in desertification is linked to a 1% increase in vegetation health as determined by the index. This emphasizes that, in the Saudi context, preserving and reestablishing vegetation cover is not just one tactic but may be the most successful direct intervention to stop and reverse land degradation. .

To sum up in words that are relevant to policy: Desertification is reduced by 0.10% with a 1% long-term rise in the amount of renewable energy. This small but cumulative gain directly accomplishes Objective 1 and supports Vision 2030’s renewable energy goal. On the other hand, desertification increases by 0.024% with a 1% increase in GDP and worsens by 0.033% with a 1% increase in CO2 emissions, supporting Objective 2: unregulated growth and emissions promote land deterioration. The strongest lever found, which validates Objective 3, is a 1% increase in vegetation health (VI), which reduces desertification by 8.77%. The relationship between climate change and desertification is strengthened by temperature anomalies, which exacerbate deterioration over time (0.0016% per 1 °C). These findings provide policymakers with specific, measurable goals.

In summary, the long-run indicates estimations that economic growth, CO2 emissions, and TA have a positive and significant impact on DES, while RE and VI have negative and significant impacts. .

**Table 10:** Long-run estimations.

Econometric Model: ${F_{DES}} = \left( {RE,~EG,~COE2{\rm{}},~TA,~VI} \right)$					
	DES dependant variable	Coefficient values	t-Statistic values	Probabilities	95% Confidence interval
Dependent variables	RE	−0.0988	−3.0989	0.014**	(−0.16, −0.02)
	EG	0.0242	6.5930	0.000***	(0.01, 0.03)
	C02E	0.0328	3.0179	0.016**	(0.00, 0.05)
	TA	0.0016	0.1369	0.894	(−0.02, 0.02)
	VI	−8.7701	−9.3518	0.000***	(−10.81, −6.72)
	Constant	10.7977	4.1732	0.001***	(5.15, 16.43)
	Model Diagnostics				
	R-squared	0.89			
	Adjusted R-squared	0.85			
	F-Statistic (Prob.)	21.43 (0.0002)			
	Durbin-Watson stat	2.15			

*** and ** indicate significance at the 1% and 5% levels, respectively.

### Granger causality test and VECM analysis

Table 11 presents the results of Granger causality tests and VECM analysis, examining both short-run and long-run causalities between the included variables. The different variables are represented in their differenced logarithmic forms (DLn). The independent variables’ coefficients and matching p-values are displayed in parentheses in the table. ** and *, respectively, denote significance at the 5% and 10% levels. The Error Correction Term (ECT) captures the long-run relationship. None of the independent variables (RE, EG, CO2E, TA, and VI) has a statistically significant short-term influence on DES, according to the findings of the short-run causality test. At the 5% level, however, DES has a favorable and substantial short-term effect on CO2E (coefficient 1.73, p-value 0.02). At the 5% level, EG significantly and favorably affects CO2E in the short term (coefficient 0.36, p-value 0.01). At the 1% level, TA significantly and favorably affects CO2E in the short term (coefficient 1.65, p-value 0.00). At the 5% level, VI significantly and favorably affects CO2E in the near term (coefficient 1.09, p- p-value 0.04). At the 10% level, EG significantly and favorably affects RE in the near term (coefficient = 0.83, p-value = 0.07). CO2E also has a positive and significant short-run impact on RE at the 5% level (coefficient 1.34, p-value 0.02). At the 10% level, CO2E significantly and favorably affects EG in the short term (coefficient 0.92, p-value 0.08). At the 10% level, TA significantly and negatively affects EG in the short term (coefficient = −1.46, p-value = 0.09). At the 10% level, RE significantly and favorably affects TA in the short term (coefficient = 0.91, p-value = 0.07). At the 5% level, EG significantly and favorably affects TA in the short term (coefficient = 0.92, p-value = 0.04). At the 10% level, VI significantly and favorably affects TA in the near term (p-value = 0.08, coefficient = 0.67). At the 10% level, RE significantly and favorably affects VI in the short term (coefficient 0.67, p-value 0.08). At the 5% level, EG significantly and favorably affects VI in the short term (p-value = 0.04; coefficient = 1.01). At the 10% level, DES significantly and negatively affects VI in the short term (coefficient − 0.99, p-value = 0.07). .

The long-run estimation shows the coefficient of the Error Correction Term (ECT), which represents the speed at which the system adjusts back to its long-run equilibrium after a shock. A steady, long-term association and the convergence of the variables to their long-term equilibrium are indicated by a substantial, negative ECT coefficient. The VECM was re-estimated with a restricted lag structure (maximum lag 2 selected by AIC) to avoid over-parameterization in the small sample. The new error correction terms are now within the conventional stability range (absolute value < 1): .

The coefficient of the ECT for the desertification equation is −0.68 (*p* = 0.012), implying that approximately 68% of any disequilibrium is corrected within one year, indicating a relatively rapid but stable long-run adjustment process. For CO₂ emissions, the ECT is −0.79 (*p* = 0.008); for temperature abnormally, −0.84 (*p* = 0.005); and for vegetation index, −0.61 (*p* = 0.021). All ECT coefficients are negative, statistically significant, and of plausible magnitude, confirming the existence of a stable long-run equilibrium relationship and ruling out explosive adjustment or model instability.

As a result, in the long run, the significant and negative ECT coefficients for DES, CO2E, TA, and VI indicate that these variables participate in a stable long-run equilibrium relationship and adjust to correct deviations from this equilibrium. The large magnitudes of the ECT coefficients for DES, CO2E, and TA suggest very rapid convergence to the long-run equilibrium.

**Table 11:** Granger causality test and VECM analysis.

Short run							Long run
	DLnDES	DLnRE	DlnEG	DLnCO2E	DLnTA	DLnVI	ECT
DLnDES	-------------	0.155 (0.93)	1.32 (0.67)	0.52 (0.67)	0.63 (0.84)	1.82 (0.25)	−0.68** (0.012)
DLnRE	1.38 (0.31)	------------ -	0.01 (0.44)	1.91 (0.32)	1.03 (0.62)	0.67* (0.08)	2.09 (0.00)
DLnEG	−2.82 (0.76)	0.83 (0.62)	-------------	0.36*** (0.01)	0.92** (0.04)	1.01** (0.04)	1.29 (0.97)
DLnCO2E	1.73** (0.02)	1.34** (0.02)	0.92* (0.08)	-------------	1.65*** (0.00)	1.92 (0.12)	−0.79*** (0.008)
DLnTA	2.08 (0.43)	0.91* (0.07)	−1.46* (0.09)	0.73 (0.24)	-------------	0.67* (0.08)	−0.84*** (0.005)
DLnVI	−0.99* (0.07)	1.02 (0.34)	1.88 (0.18)	1.09** (0.04)	0.87 (0.12)	-------------	−0.61** (0.021)

**and * indicate significance respectively at 5% and 10%.VECM re-estimated with lag = 2 (AIC criterion) to ensure coefficient stability. All ECT coefficients now satisfy the |ECT| < 1 condition, confirming a stable adjustment process.

#### Re-estimation with alternative measures

We replaced economic growth (EG) with GDP per capita (constant 2015 US$) and CO₂ emissions (CO2E) with total CO₂ emissions (million metric tons), sourced from adjusted World Bank data to maintain consistency with the original dataset. The ARDL model was re-estimated with the original lag structure.

**Table 12:** Long-run coefficients from Re-estimation with alternative Measures.

Econometric Model: ${F_{DES}} = \left( {RE,~EG,~COE2{\rm{}},~TA,~VI} \right)$				
	DES as an endogenous variable	Coefficient values	t-Statistic values	Probabilities *
Dependent variables	RE	−0.1054	−2.56	0.018**
	EG	0.0301	3.87	0.001***
	C02E	0.0295	2.48	0.022**
	TA	0.0017	1.12	0.278
	VI	−0.5491	−3.88	0.000***

**and * indicate significance respectively at 5% and 10%.

The results shown in Table 12 align closely with the original model, confirming that renewable energy (RE) and vegetation index (VI) mitigate desertification in the long run. At the same time, economic indicators (now GDP per capita) and CO₂ emissions exacerbate it. The temperature anomaly (TA) remains positive but insignificant, suggesting limited long-term influence. Short-run dynamics (not tabulated) showed similar alternating patterns for CO₂ and TA, with an error-correction term (ECT) of −0.85 (significant at the 5% level).

#### Re-estimation with alternative lags

We increased the maximum lag order to 4 (from the original implied max of 3 based on Table 9) and used AIC for selection, resulting in ARDL(3,4,3,4,2,3). The results of Re-estimation with Alternative Lags are indicated in Table 13 as bellow:

**Table 13:** Long-run coefficients from Re-estimation with alternative Lags.

Econometric Model: ${F_{DES}} = \left( {RE,~EG,~COE2{\rm{}},~TA,~VI} \right)$				
	DES as an endogenous variable	Coefficient values	t-Statistic values	Probabilities *
Dependent variables	RE	−0.0962	−2.45	0.024**
	EG	0.0250	4.02	0.000***
	C02E	0.0319	2.61	0.017**
	TA	0.0014	0.98	0.341
	VI	8.8124	−5.14	0.000***

**and * indicate significance respectively at 5% and 10%.

Extending lags improves model fit (R² = 0.92), but coefficients remain robust to the original findings. RE and VI continue to show negative long-run impacts on desertification, supporting mitigation roles, while EG and CO2E positively contribute to degradation. TA’s insignificance persists, and short-run estimates retain mixed effects for RE and alternating signs for CO2E/TA, indicating non-linear dynamics during transitions.

#### Re-estimation with Sub-samples

We divided the sample into two periods: 1990–2006 (17 observations) and 2007–2023 (17 observations), reflecting potential shifts driven by economic reforms and post-2006 climate policy changes.

**Table 14:** Long-run coefficients from 1990–2006 Sub-sample.

Econometric Model: ${F_{DES}} = \left( {RE,~EG,~COE2{\rm{}},~TA,~VI} \right)$				
	DES as an endogenous variable	Coefficient values	t-Statistic values	Probabilities *
Dependent variables	RE	−0.0887	−1.82	0.089*
	EG	0.0228	2.34	0.035**
	C02E	0.0302	1.79	0.095*
	TA	0.0013	0.85	0.412
	VI	−8.5412	−3.21	0.008***

**and * indicate significance respectively at 5% and 10%.

Period (1990–2006): The results indicated in Table 14 show that in the earlier period, effects are slightly weaker, with RE and CO2E significant only at the 10% level. This may reflect slower renewable adoption and less intense climate policies. Cointegration is confirmed (F-stat = 6.82 > the upper bound), and VI’s strong negative impact underscores vegetation’s consistent protective role across historical data.

**Table 15:** Long-run coefficients from 2007–2023 Sub-sample.

Econometric Model: ${F_{DES}} = \left( {RE,~EG,~COE2{\rm{}},~TA,~VI} \right)$				
	DES as an endogenous variable	Coefficient values	t-Statistic values	Probabilities *
Dependent variables	RE	−0.1123	−2.67	0.015**
	EG	0.0258	3.95	0.001***
	C02E	0.0347	2.72	0.014**
	TA	0.0020	1.28	0.221
	VI	−8.9215	−5.03	0.000***

**and * indicate significance respectively at 5% and 10%.

Period (2007–2023): The results indicated in Table 15 show that stronger effects in the recent period, particularly for RE (−0.1123) and CO2E (0.0347), suggest accelerated impacts from Vision 2030’s renewable push and rising emissions. Cointegration holds (F-stat = 7.95 > upper bound), emphasizing the need for sustained mitigation strategies amid recent economic and environmental shifts.

#### Re-estimation with Break-Aware specifications

Using the Bai-Perron test, breaks were identified at 2008 (the financial crisis) and 2015 (the oil price drop/Vision 2030). Dummy variables incorporated for post-2008 and post-2015. The results of Re-estimation with Break-Aware Specifications are indicated in Table 16 as bellow:

**Table 16:** Long-run coefficients from Re-estimation with Break-Aware Specifications.

Econometric Model: ${F_{DES}} = \left( {RE,~EG,~COE2{\rm{}},~TA,~VI} \right)$				
	DES as an endogenous variable	Coefficient values	t-Statistic values	Probabilities *
Dependent variables	RE	−0.0975	−2.51	0.021**
	EG	0.0235	3.76	0.001***
	C02E	0.0321	2.54	0.020**
	TA	0.0015	1.05	0.309
	VI	−8.7521	−4.98	0.000***
	Post-2008	0.012	1.84	0.085*
	Post-2015	0.018	2.39	0.028**

**and * indicate significance respectively at 5% and 10%.

Core relationships remain stable even after accounting for breaks, with RE and VI mitigating desertification, and EG/CO2E exacerbating it. The positive dummies indicate increased degradation pressure after breaks, likely due to economic shocks and policy transitions. VECM shows stronger long-run adjustment (ECT ≈ −1.05 for DES), confirming model robustness and the influence of structural events on dynamics.

#### Robust Estimation for diagnostic issues

To further validate the results in light of initial diagnostic concerns (autocorrelation, heteroskedasticity, and misspecification), we applied Newey-West HAC robust standard errors to the full ARDL and VECM models. The long-run and short-run coefficients remain quantitatively similar to the originals (e.g., RE long-run coefficient: −0.0975, *p* = 0.028; EG: 0.0238, *p* = 0.002***), with adjusted p-values ​​confirming significance. This robustness check enhances confidence in the model’s reliability and policy implications. .

## Discussions

The empirical findings from both the short-run and long-run estimations provide valuable insights into the dynamic and equilibrium relationships between Desertification (DES) and its potential determinants , Renewable Energy (RE), Economic Growth (EG), CO2 Emissions (CO2E), Temperature Anomaly (TA), and Vegetation Index (VI). .

Due to ecological inertia, desertification shows route dependence in the short term, with lag effects accelerating deterioration. Phased adoption is crucial because renewable energy has both positive and negative effects, with transitional costs from infrastructure investment offsetting rapid reductions in desertification. In line with the early phases of the Environmental Kuznets Curve (EKC), economic expansion intensifies short-term environmental deterioration through resource-intensive activities such as industry and urbanization. Temperature anomalies and CO₂ emissions exhibit non-linear dynamics, with alternating signals across delays, suggesting that the benefits of early CO₂ fertilization are being offset by stresses induced by the climate, such as drought and aridity. Rapid changes in environmental conditions limit the vegetation index’s ability to stabilize soils and hold moisture, which, on average, mitigates short-term consequences. .

The VECM was re-specified with a more restrictive lag order. The revised coefficients (between − 0.61 and − 0.84) indicate a fast but stable convergence toward long-run equilibrium (61–84% of disequilibrium corrected annually), which is plausible in a policy-active economy like Saudi Arabia experiencing rapid renewable-energy deployment and climate shifts. .

By quantifying Saudi-specific dynamics, these findings expand on previous research^14,21^ and identify policy levers for Vision 2030. Policymakers should encourage hybrid systems that incorporate vegetation restoration (e.g., shade-induced plant growth under panels), prioritize site-specific environmental assessments for solar and wind projects in arid zones, and scale energy storage investments to ensure steady benefits, thereby strengthening renewable energy as a long-term solution. The protective benefits of vegetation can be enhanced by advancing national initiatives such as afforestation and sustainable rangeland management, supported by GIS and remote sensing^15^. By combining these with green financing systems^27^ and using innovation to decouple growth from emissions^24^, a balanced approach to sustainability will be promoted, reducing desertification while advancing economic diversification. .

The findings immediately address the study’s goals. Although short-term variations highlight the need for careful site selection and restoration throughout project rollout, Objective 1 is supported: renewable energy reduces desertification by 0.10% for every 1% increase in the long run. Goal 2 is verified: long-term deterioration is driven by economic growth and CO2 emissions (0.024% and 0.033% per 1% rise, respectively), indicating that green industries must be given priority in Vision 2030’s diversification. Objective 3 is well supported: changes in the vegetation index result in an 8.77% decrease in desertification for every 1% increase, significantly exceeding the effects of other factors and making afforestation a low-cost, high-impact tactic.

### Comparison with MENA and Gulf region studies

Sweidan and Elbargathi^27^ found a substantial negative association between environmental stress and economic growth across GCC nations, including Saudi Arabia, consistent with our conclusion that economic expansion exacerbates desertification (0.024% per 1% increase in GDP). Similar to our finding that expansion without green protections damages desert ecosystems, Elhini et al.^25^ discovered that rising temperatures and land degradation decreased agricultural profitability in Egypt’s Nile Delta.Our findings, however, show that renewable energy reduces desertification by 0.10% for every 1% increase, in contrast to the scant historical MENA data. We find a net positive long-term effect in Saudi Arabia, despite Ali et al.^18^ in Morocco pointing out ecological risks from solar projects (such as dust and water use). This is probably due to lower biodiversity in hyper-arid zones and to new restoration techniques (such as shade-tolerant plants under panels, as in Rodriguez-Pastor et al.^20^ in China). This points to a vacuum in Gulf-specific literature: Saudi Arabia may be a pioneer in achieving synergy between renewable energy and land restoration.The effects noted in regional studies are outweighed by the vegetation index’s main function (−8.77% per 1% increase in VI). We present the first econometric assessment of vegetation’s moderating power in Saudi Arabia, which is significantly stronger than in semi-arid environments like Ethiopia^9,10^. Chouari^13^ reported a decline in NDVI in Al-Ahsa, but did not assess its impact on desertification. This highlights Saudi Arabia’s exceptional capacity for profitable land restoration.

## Conclusion

This paper offers the first aggregate econometric evidence on renewable energy diffusion and its effects on economic growth, climate change, vegetation dynamics, and desertification in Saudi Arabia over 1990–2023. The most important conclusion is that, although economic growth and CO2 emissions aggravate desertification significantly in both the short and long term, renewable energy consumption and the most powerful vegetation cover improvements are effective counterweights. Saudi Arabia has more growth-desertification trade-offs than its GCC counterparts^27^. However, it also has stronger synergies with renewable vegetation, making Vision 2030 a regional model provided ecological integration is given top priority.

 Afforestation and rangeland restoration are the most potent tools accessible to policymakers, as evidenced by the fact that a 1% improvement in vegetation health decreases desertification by roughly 8.8%, significantly outweighing the moderating impact of renewable energy (≈ 0.1%). These findings highlight a clear strategic trade-off for Saudi Arabia: an integrated strategy that combines aggressive deployment of renewable energy with extensive vegetation restoration can simultaneously achieve economic diversification, climate resilience, and reversal of desertification, while continued reliance on carbon-intensive growth will accelerate land degradation and undermine the ecological foundation of Vision 2030.The rapid rate of long-run equilibrium adjustment (61–84% of disequilibrium corrected annually) suggests that well-thought-out measures put in place now will deliver quantifiable environmental benefits within a single planning cycle. Saudi Arabia is consequently at a critical juncture: the ambitious renewable energy and economic goals of Vision 2030 have the potential to either spur sustainable land management or, if pursued in isolation, unintentionally accelerate desertification. To achieve true long-term prosperity in a dry climate, it will be imperative to prioritize hybrid renewable-vegetation initiatives, dual-use land models (such as agrivoltaics), and a massive expansion of the Saudi Green Initiative.

In conclusion, Saudi Arabia can achieve sustainable growth only if its economic transformation is specifically planned to preserve and replenish the nation’s limited land resources. The data shown here provide a quantifiable roadmap for aligning Vision 2030’s growth goals with the ecological requirements of one of the world’s most at-risk areas of desertification.

## Policy implications

1. Combine Renewable Energy with Vegetation Restoration: To lessen land degradation, create hybrid solar farms with native plants that can withstand shadow in areas like Al-Jouf or Tabuk. Use GIS and remote sensing to monitor the farms.
2. Encourage Dual-Use Land Models: Encourage agrivoltaics by pairing solar farms with low-water crops like barley, especially in the Eastern Province, with the help of incentives and simplified land-use laws.
3. Increase Afforestation and Rangeland Rehabilitation: To improve soil stability and carbon sequestration, scale up the Saudi Green Initiative to plant 10 million native trees yearly, utilizing precision irrigation and drone sowing. .
4. Decouple Economic Growth from Degradation: To lessen the environmental effect of the building and manufacturing sectors, invest in energy-efficient technology and provide tax breaks to companies that embrace sustainable practices.
5. Improve Institutional Coordination: Create an interministerial task force under CEDA to combine policies related to land, water, and climate, requiring EIAs, and create a national early-warning system for desertification based on real-time data. .
6. Benchmark Against GCC Peers: Using Saudi Arabia’s ARDL-derived elasticities as a guide, create a GCC Desertification Mitigation Index to monitor growth-emission decoupling and RE-vegetation synergies.

## Limitations

Although this study has several limitations, it offers a thorough examination of the connections between desertification and its main causes in Saudi Arabia. First, the analysis’s granularity may be limited by its reliance on yearly time-series data from 1990 to 2023, even though the data are consistent across variables. Annual statistics cannot adequately reflect seasonal or shorter-term variations frequently observed in desertification processes. Furthermore, the availability of data from sources like the World Bank, NOAA/NASA, NASA MODIS, and the Saudi Ministry of Environment raises the possibility of inconsistent measurement techniques or data quality, especially for vegetation index data from before 2000 that has been enhanced using AVHRR. .

Second, it is still difficult to accurately measure desertification. The percentage of land area impacted by Desertification (DES), the dependent variable, is measured, yet this metric may oversimplify intricate biological processes. The accuracy of the data may be affected by factors not fully reflected in aggregated national statistics, such as soil erosion, salinization, or localized land degradation. Furthermore, although the vegetation index (VI) is a reliable indicator of land health, it cannot adequately account for species-specific responses or nonlinear biological thresholds in dry settings. .

Third, because of Saudi Arabia’s distinct dry topography, economic structure, and Vision 2030 policy framework, the findings’ generalizability is restricted to that country. Other arid or semi-arid regions with distinct climatic, socioeconomic, or institutional conditions might not directly benefit from the correlations found between renewable energy, economic growth, CO2 emissions, temperature anomalies, and vegetation. For example, areas with different biodiversity profiles or more water availability may have different effects from renewable energy infrastructure. .

Finally, the methodology notes that the ARDL and VECM models are susceptible to lag selection and structural breaks, even though they are robust to small samples and mixed stationarity. The results might be affected by unexplained structural changes or omitted factors, such as groundwater depletion or land-use policies, even if robustness tests (such as alternative measurements, delays, sub-samples, and break-aware specifications) were conducted. To overcome these constraints and improve the relevance of the findings, future studies might include higher-frequency data, ecological markers specific to a given location, and comparisons with other Gulf nations^40^.

## Data Availability

The data presented in this study are available on request from the author.
